# A Comprehensive Review of the Study and Development of Microcapsule Based Self-Resilience Systems for Concrete Structures at Shenzhen University

**DOI:** 10.3390/ma10010002

**Published:** 2016-12-22

**Authors:** Ning-Xu Han, Feng Xing

**Affiliations:** 1 Guangdong Provincial Key Laboratory of Durability for Marine Civil Engineering, Shenzhen University, Shenzhen 518060, China; nxhan@szu.edu.cn; 2 College of Civil Engineering, Shenzhen University, Shenzhen 518060, China

**Keywords:** microcapsule, self-resilience, physical trigger, chemical trigger, bacteria, self-healing, self-recovery, performance, degradation, concrete structures

## Abstract

A review of the research activities and achievements at Shenzhen University is conducted in this paper concerning the creation and further development of novel microcapsule based self-resilience systems for their application in concrete structures. After a brief description of pioneering works in the field starting about 10 years ago, the principles raised in the relevant research are examined, where fundamental terms related to the concept of resilience are discussed. Several breakthrough points are highlighted concerning the three adopted comprehensive self-resilience systems, namely physical, chemical and microbial systems. The major challenges regarding evaluation are emphasized and further development concerning self-resilience in concrete structures will be addressed.

## 1. Introduction

Being a typical composite material, modern concrete (mostly reinforced by steel) has already been used as a construction material in civil engineering for more than a century, and it is believed to do so for another century because of the widespread availability of its constituents, and its cost-effectiveness, versatility, durability and adaptability. However, there are two major issues that raise serious concern among civil engineers worldwide [1,2], i.e., issues related to its durability and sustainability.

The first one is related to the performance degradation of a concrete structure under environmental actions. Over the years, there has been a growing understanding that all concrete structures deteriorate over time. Deterioration will change the performance of a concrete structure under various actions. Numerous forms of performance degradation have been observed in concrete structures due to the attack of corrosive ions, gases and other types of aggressive actions in the marine environment. When deterioration occurs, expensive repairs and maintenance have to be undertaken in order to maintain an adequate level of performance and the service life of concrete structures. It has been estimated that the annual cost of repair of concrete structures in Europe is in excess of $20 billion [3]. In China, the loss due to corrosion of concrete reinforcement is about 250 billion RMB per year, with the rate of loss increasing by about 10% annually.

Due to the shortage of natural resources, the increase in population and deterioration of environmental conditions, the second issue concerning sustainability emerges. It is often the case that the large-scale demolition of obsolete structures and their replacement with new ones are out of the question either due to economic reasons or a shortage of resources [4].

In recent years, so-called self-healing materials have been exploited in civil engineering, which have the built-in capability to repair structural damage autogenously or with the minimal help of an external stimulus [5,6]. By application of this new type of advanced material in civil engineering, the necessity of repair and long-term maintenance of concrete structures can be reduced or even eliminated, resulting in a durable and sustainable built environment.

A microcapsule based novel intelligent resilience system for concrete structures has been recently established and further developed at Shenzhen University. Its short history and development will be briefly reviewed in this paper. Starting from the principles of self-resilience, the logical path of development of such a system is examined. Fundamental terms related to the resilience concept and strategy are discussed. Remarkable breakthroughs regarding material systems, design procedures and numerical models are highlighted. The major challenges and further development concerning self-resilience in concrete will be addressed.

## 2. Pioneer Work Since 2008

### 2.1. Microcapsule Technology

Since 2008, attempts have been made to introduce a novel microcapsule based self-healing system for concrete at Shenzhen University [7,8,9,10,11]. The first challenge concerning microcapsule technology is to solve the contradictory point regarding the fundamental performance requirements of a microcapsule in concrete: namely, a microcapsule should be strong enough to resist mechanical impact without breaking during mixing of concrete, whereas it must not be too strong for a physical trigger (cracking) to work effectively. The second challenge is to find a suitable healing material with good fluidity and low viscosity for proper release. The third one is to ensure that the healing material can efficiently solidify as the target position is reached. 

**Figure 1:**
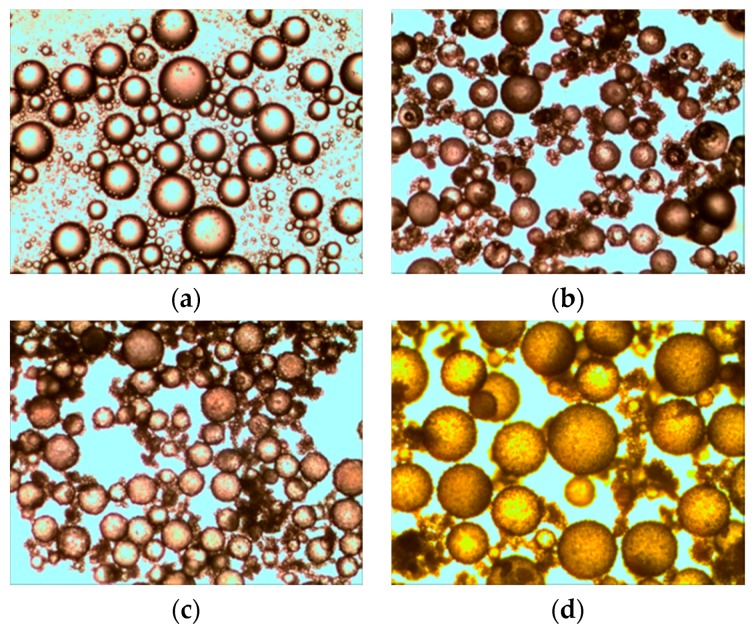
Synthesis stages of microcapsules. (**a**) Emulsion phase; (**b**) Acidification phase; (**c**) Shell forming phase and (**d**) Curing phase [7,9].

**Figure 2:**
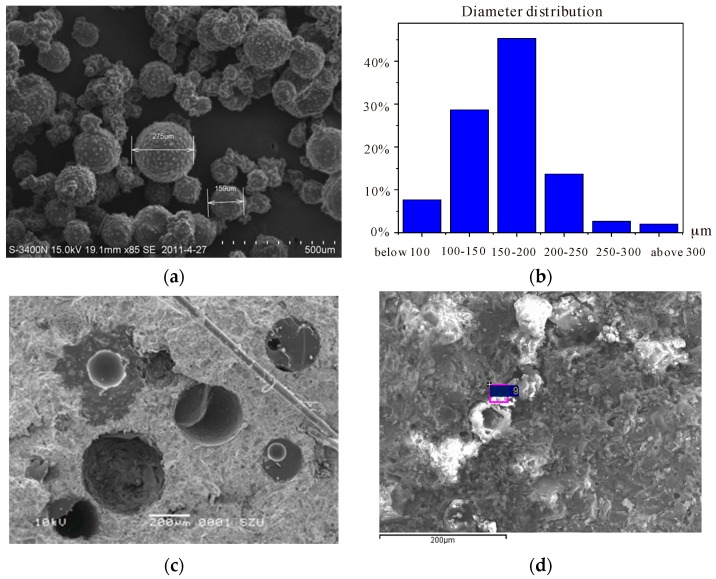
(**a**) Microcapsules obtained with the adopted production process; (**b**) Size distribution of microcapsules; (**c**) Broken surface of concrete with microcapsules; (**d**) Healing materials in cracks after solidification [9,10,11].

The urea formaldehyde resin was used for the wall of the microcapsule, and bisphenol—an epoxy resin E-51 diluted by n-butyl glycidy ether (BGE)—was adopted as the healing-agent inside the microcapsule. A combination of latent curing agent MC120D and tetraethylene penamine (TEPA)—a type of liquid curing agent functioning at normal temperature was used for curing the healing product. There were basically four stages in forming microcapsules as shown in Figure 1 (the details of the synthesis can be found in reference [7,9]):

Results showed that the microcapsule obtained with the adopted production process can be used for the self-healing system in concrete. Figure 2a shows that the shape of microcapsules is nearly regularly spherical. Nearby, small amounts of remaining epoxy were also seen, which are irregular and dispersed around microcapsules. The surfaces of microcapsules are complete and dense. There are a lot of microspherical objects on the surfaces of microcapsules, resulting in a good interface with the cement hydration products. A typical diameter distribution of microcapsules is shown in Figure 2b. The average diameter was 166 μm, and the standard deviation was 47 μm. The maximum and minimum values were 309 and 73 μm, respectively.

In the broken specimen of concrete after hardening, it could be clearly seen that the microcapsules remain undamaged during the producing process (see Figure 2c). The trigger does work efficiently (parts of the microcapsule are broken). The healing mechanism is further demonstrated in Figure 2c. The healing materials fill in the cracks and solidify afterwards. The healing mechanism is further demonstrated in Figure 2d. The healing materials fill in the cracks and solidify afterwards.

**Figure 3:**
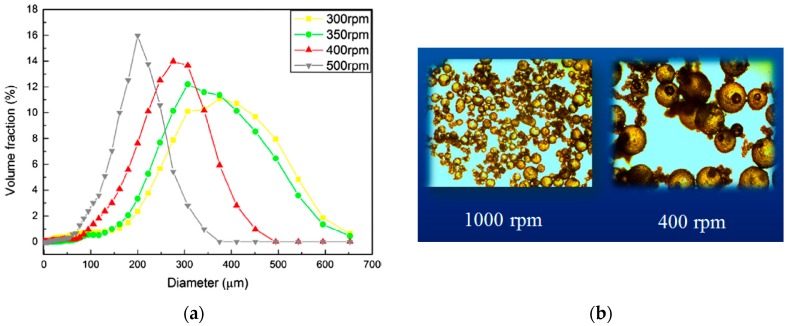
(**a**) Size distribution of the microcapsules prepared using various stirring rates [16]; (**b**) Size variation by means of two different stirring speeds [9].

It was found that the diameter, shell thickness, and surface texture of the microcapsules play major roles regarding the performance of self-healing system [7,9,10,11,12,13,14,15,16,17,18]. Therefore, the control of these key parameters during the synthesis process is critical. The average diameter and the size distribution of microcapsules can be manipulated by varying the stirring/agitation rate during the polymerization process (see Figure 3). A possible reason for this can be ascribed to the fact that the higher agitation rate provides a larger shearing force which facilitates a good dispersion of oil drops and further prevents agglomeration. This was further verified by recent studies [16,17].

**Figure 4:**
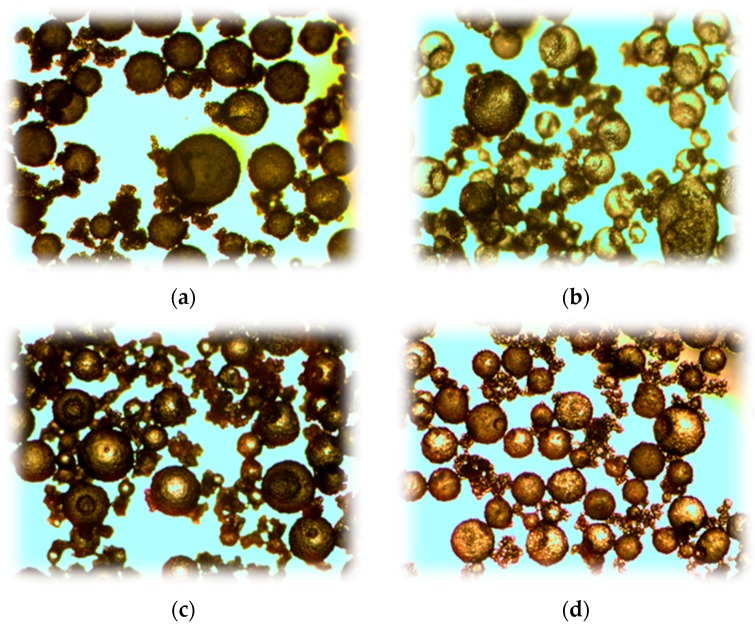
Effect of synthesis temperature on the surface texture: (**a**) 40 °C and (**b**) 60 °C; Influence of core/shell ratio on the wall thickness of microcapsule: (**c**) 0.8:1.0 and (**d**) 1.2:1.0 [7,9].

The shell thickness and the surface texture of microcapsules can be controlled by varying core/shell ratio and synthesis temperature, respectively. This is illustrated in Figure 4.

On the basis of the fundamental understanding of characteristics of microcapsules and their influences on the performance of a self-healing system, further optimizations and improvements have been made since 2008 at Shenzhen University. One way is to narrow the size distribution in order to improve the efficiency of the physical trigger [19]. Besides, microcapsules with a narrow size distribution can offer many other benefits, including tight control of the release rate of the core material. In addition, alternative healing materials were experimented with in order to improve their dispersion performance [20].

### 2.2. Feasibility Study and Evaluation

The healing efficiency with respect to the mechanical and permeability related performance of the adopted self-healing system in concrete was evaluated by experiments [7,8,9,10,11,12]. Several important parameters had been studied, such as the size and dosage percentage of microcapsules, loading level, temperature, etc. 

**Figure 5:**
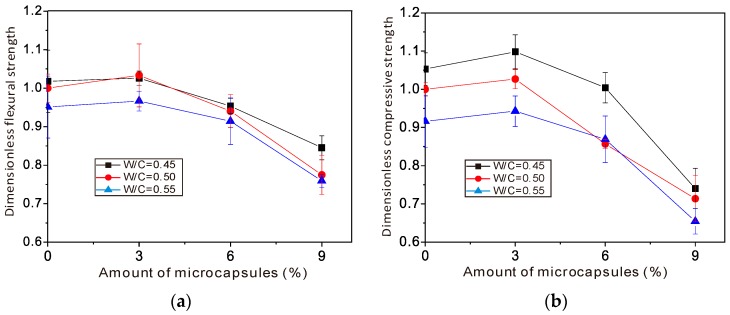
The influence of dosage percentage of the microcapsule on the mechanical behavior of concrete: (**a**) flexural strength and (**b**) compressive strength [10,11].

As shown in Figure 5, there is a tendency for the mechanical strength of concrete to reduce as the dosage of microcapsules increases. However, the impact can be neglected as the dosage of microcapsule is about 3%, which may be regarded as an optimum value.

The two different healing mechanisms were evaluated, namely mechanical performance recovery (healing) and permeability related performance recovery. Details of evaluation can be found in references [9,10,11,12].

**Figure 6:**
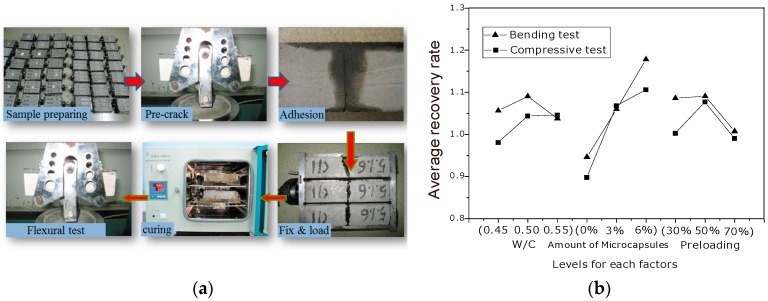
(**a**) Test procedure concerning the recovery evaluation of mechanical performance of the self-healing system in concrete; (**b**) Influences of various factors on the recovery level [10,11].

From Figure 6 it can be seen that the average recovery rate is almost proportional to the dosage of microcapsules (recovery rate = performance index after healing/original performance index), whereas there is no substantial influence of the other considered factors (preloading and W/C) on the strength recovery.

**Figure 7:**
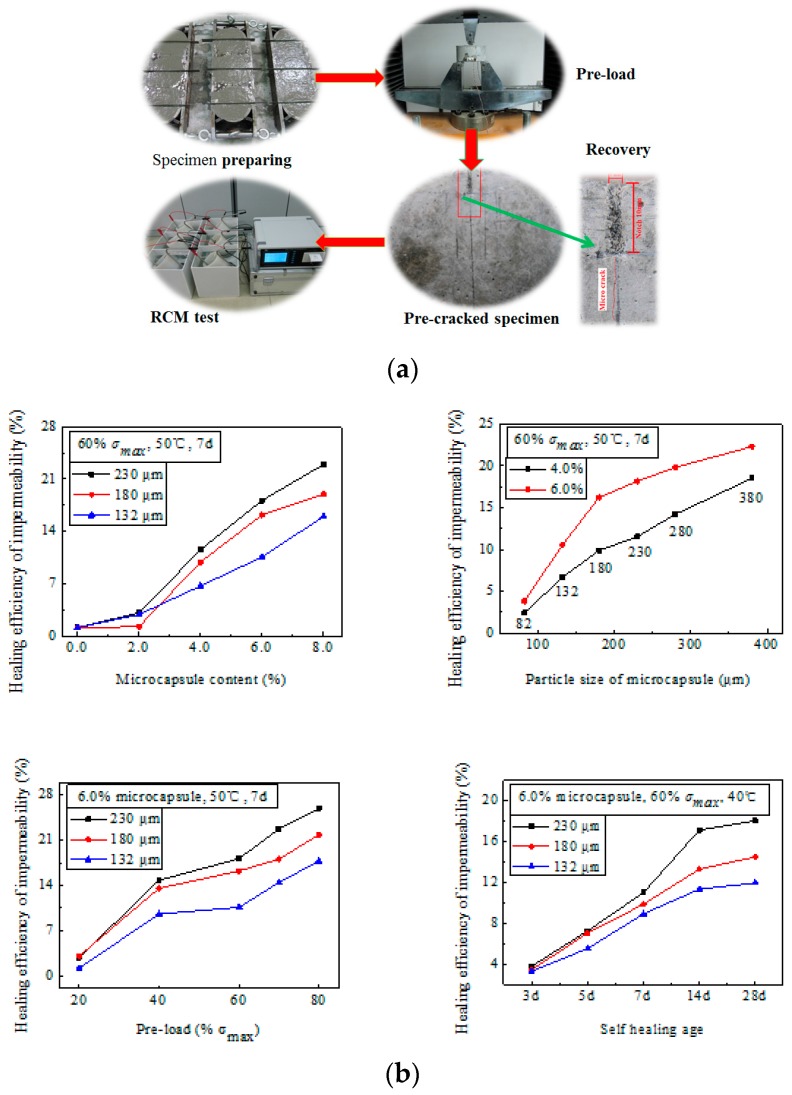
(**a**) Test procedure concerning the recovery evaluation of permeability related performance of the self-healing system in concrete; (**b**) Influences of various factors on the recovery level [10,11,12].

Permeability related recovery was evaluated by means of the RCM (Rapid Chloride Migration) test. The recovery is calculated as ((*D*_afterpreloading_/*D*_healed_) − 1) × 100%, where *D*_afterpreloading_ and *D*_healed_ are the diffusion coefficient obtained after the preloading and after healing, respectively. The tendency of recovery shows a linear increase for the adopted parameters, such as the dosage, size of microcapsules and the pre-loading level, whereas the healing temperature demonstrates as strong an effect on the recovery (see Figure 7).

## 3. Principle and Strategy Development

It is clearly shown that attempts to introduce a novel self-healing system in cementitious composites at Shenzhen University have been successful. This encouraged the research team to further explore a more comprehensive system for concrete structures.

On the basis of research and application experience concerning the service life of concrete structures, in particular, in marine environments, the required fundamental performances (at three different levels: namely material, element and structural level) are classified and it is clear that the degradation of performance of a concrete structure starts at the material level. Anything related to durability should be implemented at this point. Therefore, degradation-related resilience is paramount and forms the foundation for the strategy development of the targeted self-resilience system [2,4,21].

**Figure 8:**
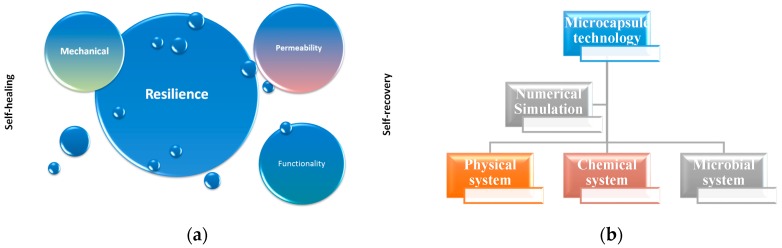
(**a**) Principle of resilience; (**b**) Strategy for development of a microcapsule based comprehensive self-resilience system for concrete structures [20].

The principle of resilience consists of two important aspects (see Figure 8a): namely, healing (related to mechanical damage) and recovery (related to the recovery of functionality).

It has to be emphasized that the self-resilience system is based on the microcapsule technology. In other words, the container of healing materials is restricted to the microcapsule, however, the type of materials of microcapsules could be essentially different (e.g., organic or in-organic, life-friendly, water-proof, etc.), depending on the targeted recovery performance index.

As far as the healing-agent is concerned, there are a large range of options, from organic, bacteria to in-organic materials.

With respect to the trigger, not only is the physical trigger (mechanical trigger) further explored, but the so-called chemical trigger has drawn major attention in our current research. This is in turn related to the resilience principle, namely, healing and recovery at a multi-scale.

In summary, three relatively independent but strategically integrated approaches have been adopted aiming at the formation of a microcapsule based comprehensive self-resilience system. The targets are to heal the mechanical damage or to recover lost functionality corresponding to impermeability or corrosion protection (Figure 8b):

- Physical system (organic or in-organic healing agent, physical trigger, self-healing at micro or meso level)
- Chemical system (organic or in-organic healing agent, chemical trigger, self-recovery at multi scale)
- Microbial system (bacteria as healing agent supplier, physical trigger, self-recovery at meso or macro level)

## 4. Several Major Breakthroughs

Relying on several cohesive research projects funded by the Nature Science Foundation of China, several breakthroughs have been made recently at Shenzhen University. They will be briefly highlighted in the following section.

### 4.1. A Chemical System with a pH Sensitive Trigger

**Figure 9:**
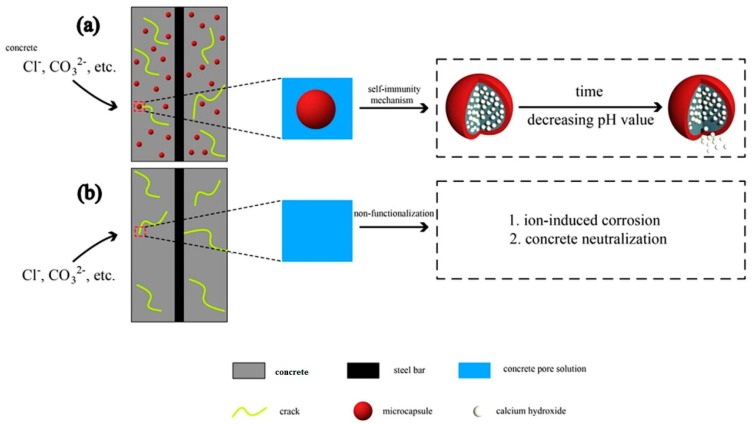
The working principle of a microcapsule based self-recovery system with a pH sensitive trigger for the protection recovery of rebars against corrosion: (**a**) system in reinforced concrete; and (**b**) situations after the trigger of the self-recovery system is excited [24].

A microcapsule based self-recovery system with a chemical trigger (in this case pH sensitive) has firstly been proposed and later on further developed at Shenzhen University [22,23,24,25,26,27], aimed at the performance recovery in protecting rebars against corrosion. The fundamental idea behind this system is outlined in Figure 9.

To prepare the microcapsules, sodium monofluorophosphate and microcrystalline cellulose were mixed into Polysorbate 80. Then, the small spherical particles were molded by the extrusion–spheronization method, which is able to control the diameter of a microcapsule. Finally, the spray drying method was adopted to fabricate microcapsules, spraying the liquid PS onto the surface of spherical particles. Three types of microcapsules with different amounts of shell materials were fabricated; the mass percentages of shell materials were 10%, 20% and 43%. For example, 10% refers the percentage of increased weight of microcapsules, according to that of spherical particles molded by the extrusion–spheronization method.

**Figure 10:**
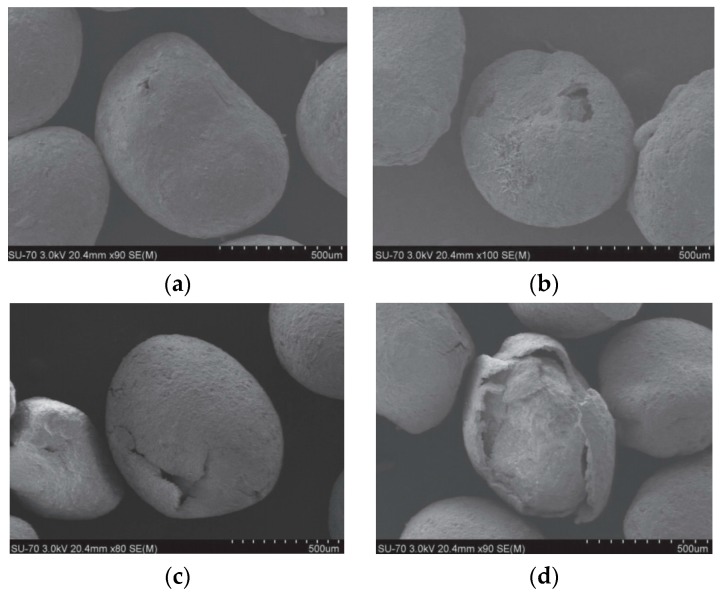
SEM images of microcapsules after soaking in solutions with different pH for the same soaking time: (**a**) pH = 13; (**b**) pH = 11; (**c**) pH = 9 and (**d**) pH = 7 [22,23].

The results of studies on the surface damage of microcapsules at different pH values by means of SEM are illustrated in Figure 10. SEM images indicate the surface changes of microcapsules after soaking in solutions with different pH values for equal periods of time. For pH = 13, there is no obvious damage on the surface of the soaked microcapsules. As the pH value is reduced, surface damage becomes more and more obvious. In a neutral environment with pH = 7, the surface of a microcapsule is totally destroyed after soaking. This further confirms that the environmental pH can be used as a trigger mechanism for this type of self-healing system.

The release mechanism is further studied after the trigger is excited [24]. To examine the release kinetics of the ethyl cellulose/calcium hydroxide microcapsules, a pH meter and a micro-plate reader were employed to monitor the variation of pH value and the release process of the capsule core, respectively. The calcium ion calibration curve of OD (optical density) produced at 575 nm is utilized to determine the amount of Ca^2+^ released from the capsule cores.

**Figure 11:**
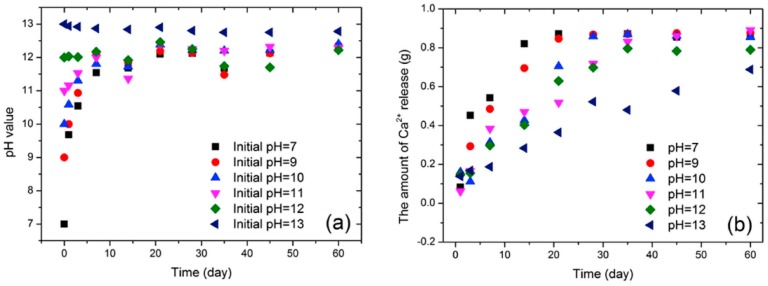
The change of pH value and the amount of Ca^2+^ with time based on the six different initial pH values: (**a**) pH—time and (**b**) the amount of released Ca^2+^—time [24].

According to the calibration curve, the amount of healing material released from the microcapsules at every designated pH level was calculated, as the pH value was measured. Figure 11a plots the change of pH value over time for different initial pH values. After a 60-day test, the pH value tended to be stable at approximately 12.5 for all adopted initial pH values, although fluctuations appeared during the middle period in some cases. The dramatic change of pH value at the beginning of the tests suggests that healing materials were released from microcapsules due to the gradual breaking down of microcapsules induced by low pH level. The accumulative amount of calcium ion released at different pH values as a function of time is illustrated in Figure 11b. The release amount significantly increased with time for all adopted initial pH values, except for the case of pH = 13 which exhibited almost no change in the considered release amount. Details over the release mechanism can be found in reference [24].

To evaluate the recovery efficiency, a self-recovery system with sodium monofluorophosphate (Na_2_PO_3_F) as the healing material was adopted. The system is triggered by pH value variation. The study focused on examining the healing mechanism of the system for chloride-induced corrosion of rebar in a simulated concrete environment [26]. The microcapsules were then put into the simulated concrete environment in order to evaluate their protection performance against steel corrosion by means of electrochemical impedance spectroscopy established in early studies [25]. 

**Figure 12:**
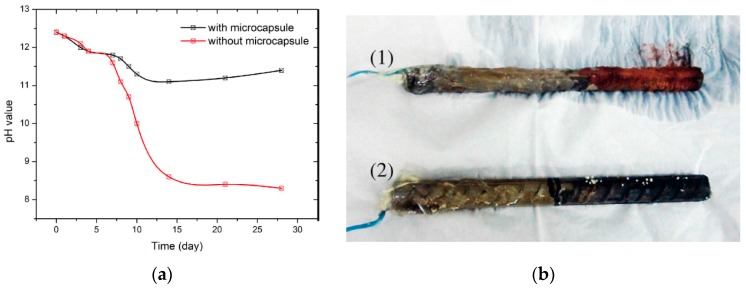
(**a**) Variation in pH value with time in saturated calcium hydroxide solutions with or without microcapsule; (**b**) rebar exposed at 3000 mg/L Cl^−^ concentration for 100 days (1) no microcapsules and (2) with microcapsules [26].

The controlled-release testing, according to the description in Ref. [24], is performed to compare the simulated concrete pore solution without any defense and the solution with microcapsules. Figure 12a demonstrates the relationship between time (day) and pH value. It seems that the two types of solution experience a constant drop in pH value. It is during the sixth day that the pH self-regulating ability of microcapsules starts to appear. Afterward, remarkable differences can be observed. Moreover, the pH value of the solution with the microcapsule slightly increases after the 14th day. This feature may be attributed to the pH-sensitive capsule which is able to regulate the concentration differences of OH between inner and outer microcapsules. However, in the case of the solution without a microcapsule, a consistent drop occurs. The same conclusion can be drawn from the sample photographs (Figure 12b) of steel rebar corrosion after 100-day exposure to Cl^−^ at a concentration of 3000 mg/L. Extensive corrosion is observed for the sample without microcapsule, in clear contrast to that with a microcapsule based sample for which no obvious corrosion is evident [26].

Very recently, a kind of microcapsule based self-recovery system with ethyl cellulose (EC)/calcium hydroxide was used to recover the protection ability against corrosion based on increasing hydroxyl content in concrete [27]. The capacity to lift the threshold value of [Cl]/[OH] in this system is evaluated. Different dosages of microcapsules are placed in the sodium chloride solution with a concentration of 3.5 wt % to characterize the corrosion protection by means of linear sweep voltammetry (LSV) and electrochemical impedance spectroscopy (EIS). The working mechanism of this system consists of the chemical triggering (pH sensitive shell) and releasing (healing material) processes when a microcapsule is exposed to a decreasing pH condition, and the release of healing materials in turn restores the alkaline environment to protect against steel bar depassivation. The test results of environmental scanning electron microscopy (ESEM) equipped with texture element analysis microscopy (TEAM) reveal that the capsule shell sensitive to the [Cl]/[OH] ratio forms an excellent low-pH trigger mechanism.

### 4.2. A Chemical System with a Chloride Sensitive Trigger

A novel idea related to the performance self-recovery was first proposed and patented by Shenzhen University. By means of a chemical trigger (chloride sensitive), a self-resilience system based on the microcapsule technology has been proposed and developed, where the capacity to resist the penetration of harmful agent (chloride ion) into concrete will be restored. As the concentration of this harmful agent reaches a certain level, the microcapsule will be broken and the healing material inside the microcapsule will take action to remove the harmful agent. A remarkable breakthrough was made in 2015 related to this [28]. Various attempts have been made since then and progress has been realized [29,30,31,32,33,34]. Up to now, various types of chloride sensitive microcapsules were obtained and further optimization is needed before this can be fully implemented in concrete. 

**Figure 13:**
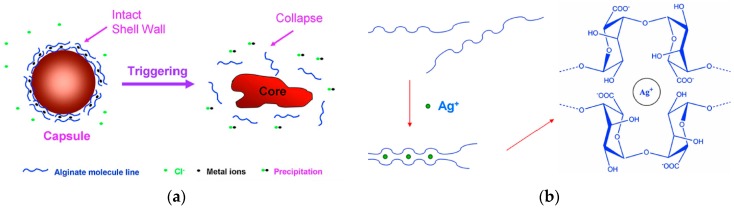
(**a**) Schematic of capsules triggered by chloride ions; (**b**) The structure of alginate chelated with Ag^+^ [28].

A chloride sensitive microcapsule was first fabricated by using a silver alginate hydrogel that disintegrates upon contact with chloride ions which provide a stable trigger for responsive microcapsules [28]. A facile method is to fabricate responsive wall materials of microcapsules containing metal ions (such as Ag^+^, Pb^2+^). When these microcapsules come into contact with chloride ions, the metal ions will be extracted to disintegrate microcapsules (see Figure 13a). Alginate is a natural anionic polymer acquired from brown algae and has been extensively utilized for biomedical applications. Sodium alginate can be cross-linked with many metal ions (such as Ca^2+^) to form hydrogel. In our first try, Ag^+^ is chosen to be coordinated with alginate to form the wall of microcapsules. Silver alginate (Ag-alg) can also form a characteristic “egg-box” structure. Each alginate molecular chain can be linked with other chains, forming a three-dimensional gel network (see Figure 13b), which is strong enough to form the wall of microcapsules.

**Figure 14:**
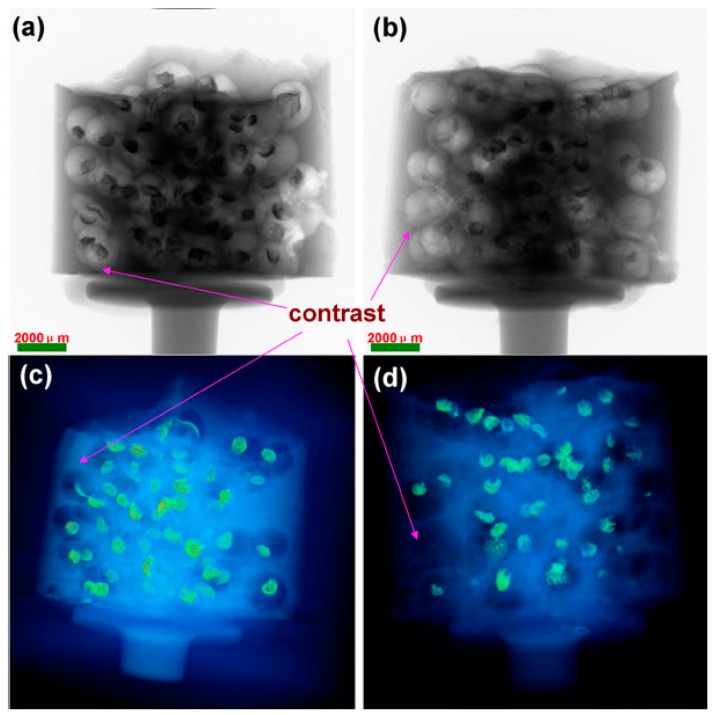
X-ray CT images of concrete: (**a**) specimen before test; (**b**) specimen soaked into NaCl solution; (**c**) 3D image of concrete specimen and (**d**) 3D image of concrete specimen soaked into NaCl solution [28].

X-ray computed tomography (X-ray CT) was employed to detect concrete specimen embedded with Ag-alg microcapsules. Figure 14a shows the concrete specimen embedded with Ag-alginate microcapsules, but is not soaked with NaCl solution. Figure 14b depicts the concrete specimen exposed to the chloride environment. It is clear that the microcapsules near the surface of concrete specimen disappeared. In X-ray CT 3D model, heavy metals like Ag have a strong absorbency for X-ray, and appear as bright objects in 3D images. In comparison with X-ray CT 3D images (Figure 14c,d), the microcapsules near the surface of concrete specimen also disappeared. This means that the adopted microcapsule system is sufficiently chloride sensitive.

### 4.3. Microbial Self-Healing System 

Self-healing of concrete cracks by bacteria is based on microbial-induced calcium carbonate (CaCO_3_) precipitation, which is a common phenomenon in the natural environment. Certain mineralization bacteria can make use of carbon source to produce CO_2_ or CO_3_^2−^, which subsequently reacts with calcium ions to form calcium carbonate precipitate on the surface of concrete cracks, thus sealing the concrete cracks. At the same time, the metabolism of mineralization bacteria creates an alkaline environment, which is further in favor of the process of calcium precipitation. Moreover, bacteria also provide nucleation sites for calcium precipitation. In addition to the ability to induce calcium precipitation, self-healing bacteria should be alkaliphilic and spore-forming due to the harsh environment inside concrete.

The proposed microbial self-healing system at Shenzhen University is a microcapsule based system. It has clear advantage in comparison with other types of microbial self-healing systems. The spore has to be untouched during mixing of concrete. In addition, it cannot unintentionally come into contact with water, which will result in premature bacteria mineralization. Therefore, it is ideal to encapsulate bacteria in microcapsules to safeguard spores and to introduce a trigger system for smart healing.

In the last few years, several tough issues have been dealt with and some of the achievements will be briefly introduced in the following sections.

#### 4.3.1. The Calcium Precipitation Activity (CPA) Related Issues

The CaCO_3_-mineralizing bacteria from different taxonomic groups have shown potential in restoration of construction material such as concretes, cements and stone materials. However, these strains do not meet the demands of practical applications due to some shortcomings, including low mineralizing capacity. 

The first step is to select the most efficient bacteria species in order to obtain a high-efficiency calcium-precipitating bacterium [35,36,37,38]. An integrated high-throughput screening (HTS) strategy was developed for the determination of calcium precipitating activity (CPA) of bacteria. The isolates, mutagenized by ultraviolet radiation (UV), were cultivated in the suitable media containing calcium ion by using 96-deep-well microliter plates. The residual calcium in supernatants from micro-cultivation plates was determined by O-Cresolphthalein Complexone method to evaluate the CaCO_3_-producing activity. On the other hand, the activity of carbonic anhydrase of the isolates, which is responsible for the formation of CO_3_^2−^, was also monitored to further evidence the bacteria-induced calcium mineralization process. It is proved that this novel HTS strategy is a promising procedure for selecting highly efficient calcium mineralizing microorganisms [35].

**Figure 15:**
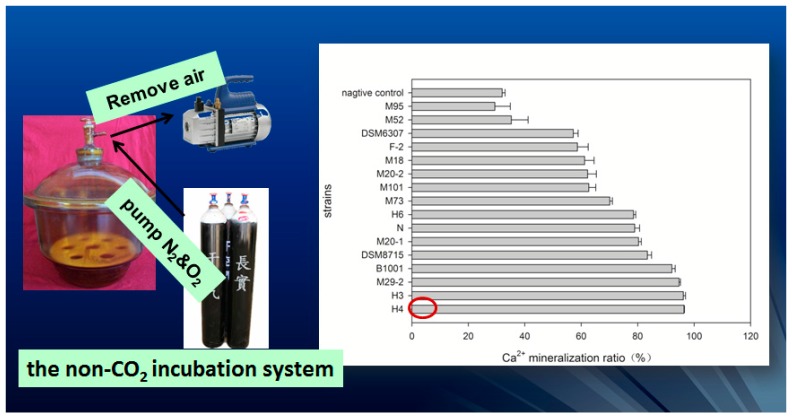
The screening of high-efficiency Ca-mineralizing bacteria [36].

In addition, 13 morphologically different strains were obtained from mangrove sediment and soda lake sediment. The calcium precipitating activities (CPA) of the strains were evaluated [36], and strainH4 (a type of bacteria strain identified as bacillus species) exhibited the highest CPA value (see Figure 15). Strain H4 was identified as bacillus species based on the 16S rDNA sequence analysis. Further, effect of variable factors on calcium precipitation of strain H4 was evaluated. The result showed that sodium lactate and sodium nitrate were the best carbon source and nitrogen source for the precipitation of calcium ion, respectively. 

**Figure 16:**
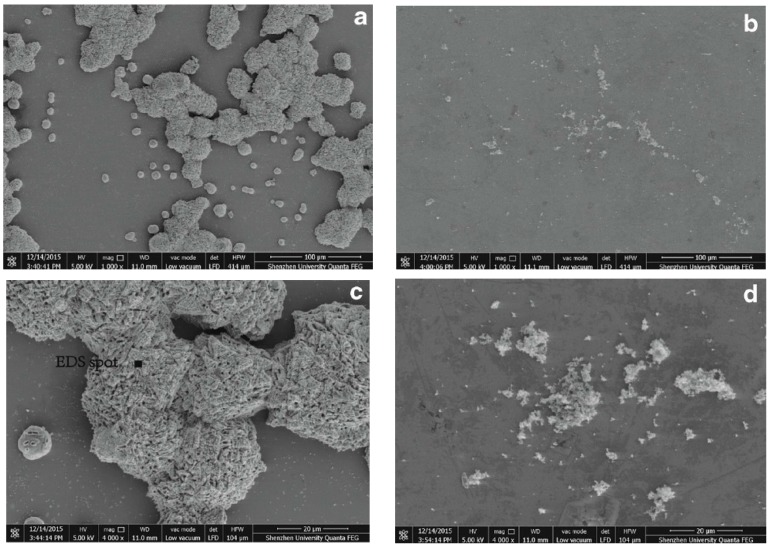
SEM images of CaCO_3_ precipitation: (**a**) bacteria-inoculated; (**b**) bacteria free; (**c**) bacteria-inoculated; and (**d**) bacteria free [37].

The features of the precipitated products were further studied [37]. As shown in the SEM images (Figure 16a,c), a large amount of crystals grew in the bacteria-inoculated medium. Two types of morphological crystals were observed, single irregular spheres and irregular branches/aggregates of spheres (Figure 16a). Both types of crystals had rough surfaces with many deformed lamellar rhombohedra (Figure 16c). Single irregular spherical crystals with a size of 20–40 μm agglomerated into irregular branches. Moreover, the crystals showed evidence of bacterial involvement. Rod-shaped and round holes (1–4 μm) were found on the surface of the crystals (Figure 16c), which presumably occurred in the space occupied by the bacterial cells or spores. These holes in the crystals also suggested that bacteria served as nucleation sites during the mineralization process. For comparison, in bacteria-free medium, only fusiform and amorphous crystals were formed, and the amount of formed crystals was much smaller than that in bacteria-inoculated medium (Figure 4b,d). Furthermore, no sign of bacteria involvement was observed during the production of crystals.

Element composition analysis via energy dispersive spectroscopy (EDS) revealed that the crystal is primarily composed of calcium, carbon and oxygen with a weight ratio closely matching that of CaCO_3_, indicating that the crystal is CaCO_3_. Furthermore, the XRD analysis confirmed that the crystal induced by H4 is calcite [37].

Influential factors in the process of bacteria-induced calcium precipitation such as the carbon source, nitrogen source, pH and Ca^2+^ concentration were also studied [37]. Experimental results revealed that sodium lactate and sodium nitrate were the best carbon and nitrogen sources for H4. Moreover, pH poses a significant impact on the bacteria-induced calcium precipitation, and H4 could effectively induce calcium precipitation in the pH range of 9.5–10.5. Although it is difficult to determine the actual pH inside a crack of concrete after ingress of water or moisture, the inner microenvironment will be alkaline to a great extent due to the existence of Ca(OH)_2_. Therefore, H4 is a promising bacterium for self-healing of concrete cracks, considering that its CPA can remain at greater than 80% even if the pH reaches 11.0. In addition, it was found that the presence of excessive Ca^2+^ not only inhibits the bacterial-induced calcium precipitation process but also results in waste of the Ca^2+^ resource, and maintenance of a Ca^2+^ concentration lower than 30 mM is a good strategy. Fortunately, due to the poor solubility of calcium hydroxide, the free Ca^2+^ concentration of the pore solution inside the concrete is normally less than 30 mM [23], making bacterial-induced calcium precipitation feasible. Furthermore, introduction of an extra Ca^2+^ source into concrete for the microbial self-healing process, as used in selected previous studies, might not be necessary from this point of view.

It was observed that there exists a major drawback in the current microbial self-healing system, i.e., the calcium precipitation seems to only happen on the concrete surface area nearby the crack. The reason why CaCO_3_ cannot be precipitated deep inside the crack might be because of the shortage of oxygen. It is well known that both water and O_2_ need to be present inside the crack to activate the bacterial self-healing concrete. While water is sucked into the tiniest microcracks due to capillary suction, the question may arise as to whether adequate oxygen will be available deep inside the cracks. Given that both bacterial spores and nutrients were provided in the self-healing system, lack of oxygen inside the concrete structure might be a major inhibition factor. 

To deal with the problem mentioned above, a pioneer attempt at Shenzhen University has been made to develop a strategy to supply oxygen for the microbial calcium precipitation performance [39]. Firstly, a suitable peroxide was screened to develop an oxygen-releasing tablet (ORT) that can provide a stable oxygen supply. Then, the effect of oxygen provided by the selected ORT on the microbial-induced self-healing process, including the germination of dormant spores and calcium precipitation activity of the bacteria, was evaluated. Furthermore, a binary self-healing system which contains bacteria and ORT was established and the overall calcium-precipitating activity of the system was investigated. Details can be found in the reference [39].

**Figure 17:**
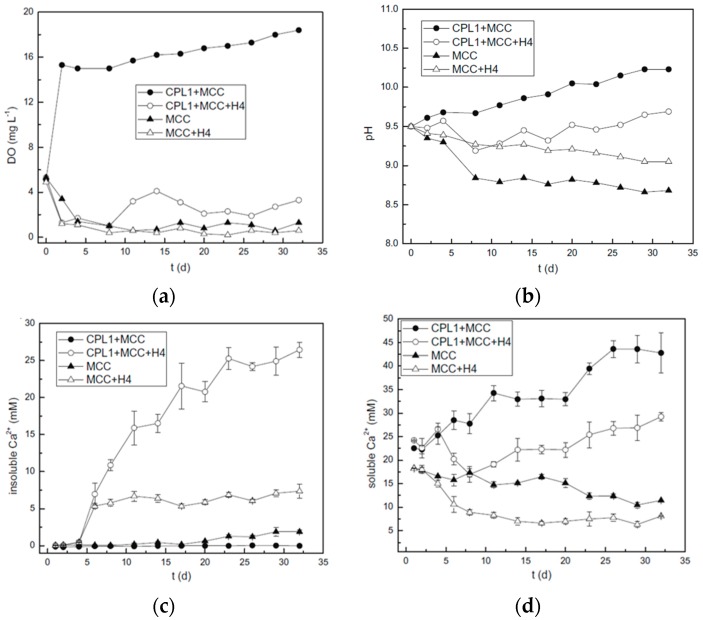
DO and pH change during calcium precipitation process of binary microbial self-healing system. MCC and CPL1 represent the microcrystalline cellulose and oxygen release compound with the ratio of CaO_2_ and lactic acid being 9:1, respectively: (**a**) DO and (**b**) pH; Calcium precipitation performance of binary microbial self-healing system. MCC and CPL1 represent the microcrystalline cellulose and oxygen release compound with the ratio of CaO_2_ and lactic acid being 9:1, respectively: (**c**) Insoluble Ca^2+^ and (**d**) soluble Ca^2+^ [39].

The calcium precipitation of the binary self-healing system was evaluated in bacteria medium solution, and the result was shown in Figure 17 [39]. 

From Figure 17, it can be seen that during the first 5 days, no calcium precipitation was observed. It might be because spores need time to germinate and grow in the vegetative cells. After 5 days, CaCO_3_ started to form in the tubes where bacteria were incubated. In the beginning stage of calcium precipitation, no difference in the formation of CaCO_3_ could be found between the tubes with or without oxygen supply. However, for the tubes with oxygen supply, the amount of insoluble Ca^2+^ increased sharply from 7 days. After 32 days, insoluble Ca^2+^ reached 27.5 mM (Figure 17c). Conversely, for the samples without oxygen supply, no visible increase of insoluble Ca^2+^ was obtained by H4 after 7 days. Only 6.9 mM of insoluble Ca^2+^ was achieved at 32 days, which is almost 25% of that with oxygen supply. Furthermore, more Ca^2+^ that was precipitated by H4 in the presence of CPL1 can be further confirmed by the result in Figure 17d that the presence of oxygen led to a smaller increase of soluble Ca^2+^ concentration than that without oxygen supply. Figure 17a exhibits the DO (dissolved oxygen) change in the process. It was clear that CPL1 provided a stable oxygen supply. The DO maintained more than 15 mg·L^−1^ during the whole process with no bacteria involved. However, the presence of H4 resulted in a huge decrease of DO from more than 15 to less than 4 mg·L^−1^. Furthermore, it can be noticed that the DO of the control (MCC/H4) was very low, which might account for the lower calcium precipitation by the bacteria in the absence of oxygen supply (Figure 17c). From Figure 17b, it can be found that involvement of the bacteria alleviated the fluctuation of pH during calcium precipitation [39].

The primary experimental results demonstrated that bacterial spores in binary self-healing system (bacteria/ORT) are able to germinate, grow, and induce calcium precipitation effectively, and the stable oxygen supply is important for microbial calcium precipitation. Further study will be related to the longevity issues of the adopted bacteria/ORT system.

#### 4.3.2. Special Issues Related to the Microcapsule Technology for Microbial Self-Healing System

Generally speaking, natural polymers are biocompatible while synthetic polymers are deleterious in various degrees for microorganisms like bacteria. That is why most of the existing biological microcapsules are walled by natural polymer materials. However, natural polymers are usually hydrophilic, i.e., they take up water and cause the shell of the microcapsule to swell. It is clear that this is not suitable for self-healing systems for concrete. 

Various attempts were made at Shenzhen University to find harmless and waterproof microcapsules which are suitable for application in microbial self-healing systems [40,41,42,43].

**Figure 18:**
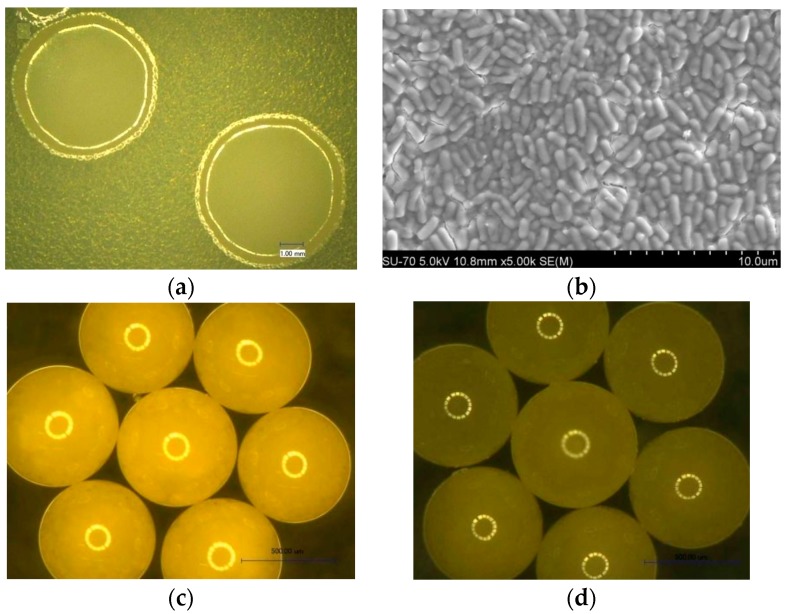
(**a**) The OM photographs of bacterial colony on the plate after 24-h cultivation; (**b**) SEM image of the bacteria in colony; (**c**) Microcapsules before soaking in water; (**d**) Microcapsules after soaking in water [40].

A typical example will be given in this section concerning the abovementioned research. In one study, Koch’s bacillus DSM6307 was encapsulated in polydimethylsiloxane with hydrophobic epoxy resin [40]. The process is anhydrous to avoid the germination of spores. All the materials used were harmless. To confirm this, a biocompatibility test was carried out by taking the same amount of spores mixed with epoxy E-51, KH-792 and water, respectively, resting for 160 min, isolating and collecting the spores which were then cultured for 24 h. Afterwards, the culture medium was extracted to determine the optical density by 490 nm-ultraviolet absorption value. OD_490_ are positively correlated with the bacterial concentration. After encapsulation, the fraction of surviving spores is over 90%, indicating the process has a mild effect on spores’ viability (see Figure 18a,b). The water tightness was tested by soaking microcapsules in water for 10 days. No change in shape, size and color of microcapsules was observed. The time was prolonged to 1 month, with no further change. So, it can be concluded the microcapsules have excellent waterproof performance (see Figure 18c,d).

#### 4.3.3. Feasibility Study on the Microcapsule Based Microbial Self-Healing System

**Figure 19:**
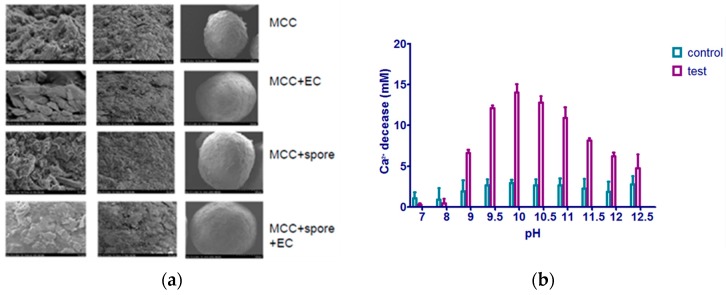

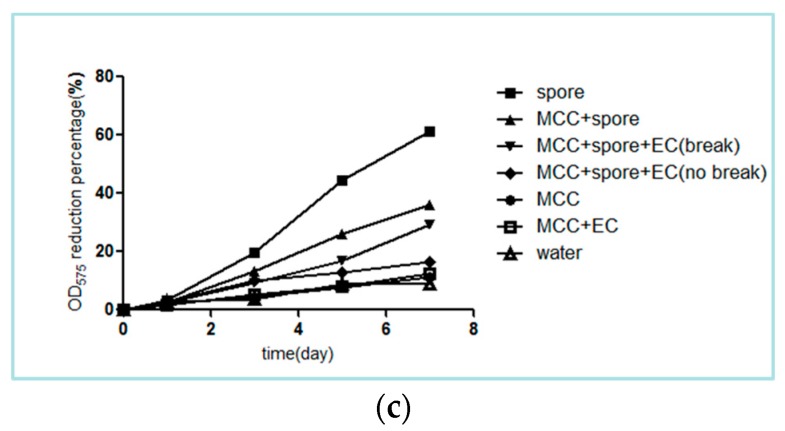
(**a**) An example of an encapsulation process for a microbial system; (**b**) The influence of pH on the bacterial mineralization; (**c**) Comparison of CPA of bacteria in different forms [45].

The realization of a real microcapsule based microbial self-healing system in concrete is regarded as an essential step towards the application of such technology in civil engineering. To prove this idea from paper to reality, it is mandatory to carry out a feasibility study. A first attempt was made to fabricate a microbial system in concrete by encapsulating microcrystalline cellulose (MCC) mixed with spores. The shell material was ethyl cellulose (EC) in the first attempt [44] and later on it was changed to a type of water-proof material [45]. The encapsulation process is illustrated in Figure 19a. The encapsulation process comprised: (a) forming the grafted skeleton by using microcrystalline cellulose (MCC); (b) mixing alkaliphilic spores with high mineralizing activity with MCC; and (c) encapsulating MCC + spores in ethyl cellulose (EC) or epoxy resin (ER).

The survivability of the encapsulated bacteria was studied, together verifying the protection functionality of the microcapsule. It was found that pure spore has the highest mineralizing activity. Broken microcapsules have higher mineralizing activity than non-broken ones, which indicates that EC microcapsules can effectively protect microorganisms in this system (Figure 19b,c).

**Figure 20:**
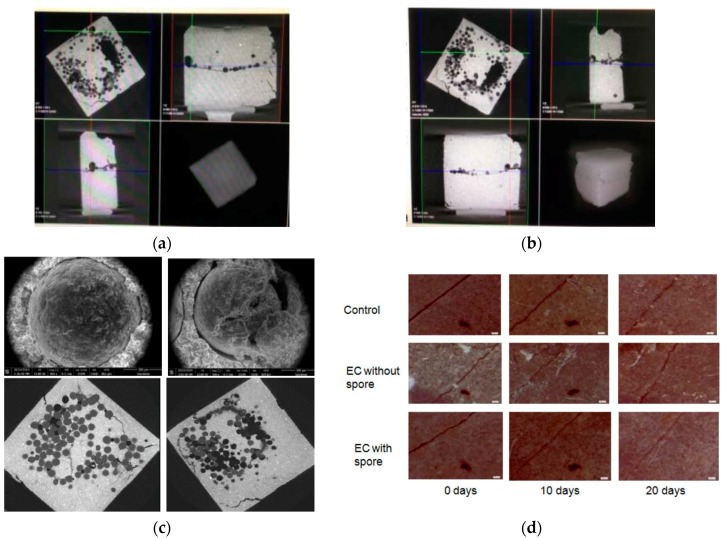
(**a**,**b**) Breakages of microcapsules upon cracking and subsequent healing procedure monitored by XCT in 3D; (**c**) SEM images and XCT results of the microcapsule before and after exciting of the trigger; (**d**) Formation of crack and subsequent healing procedure monitored by optical microscopy [45].

The trigger mechanism and the working process was further studied by means of the optical microscopy and the high resolution X-ray Computed Tomography (XCT) [44]. The crack-introduced specimen with a crack width from 20 to 50 μm was carefully studied to determine the working mechanism of the trigger. It was shown in Figure 20a–c that some microcapsules were fractured upon the formation of a crack, indicating that the desired trigger system actually works. Subsequent production of calcium carbonate confirmed by EDS indicated activation of encapsulated mineralization bacterium. The crack-healing process, mechanism and healing effectiveness were studied to evaluate the feasibility of the microbial self-healing system. Compared with the specimens without embedded bacterium, the cracks in the specimens embedded with bacterial microcapsules were largely filled (Figure 20d), which suggests that self-healing of concrete cracks can be achieved by introducing encapsulated mineralization microorganisms into concrete structures.

### 4.4. Evaluation of a Microcapsule Based Self-Resilience System

The behavior and performance of the microcapsule based self-resilience system have to be evaluated through effective and meaningful ways (either experimentally or numerically). Up to now, various techniques have been applied to appraise the obtained system with success to some extent. In addition to the traditional macro test methods (for determination of mechanical and transport properties of concrete) [26,46,47,48], the more advanced technology, such as the X-ray computed tomography (XCT) and the image analysis technique, has been adopted in the evaluation process [49]. 

**Figure 21:**
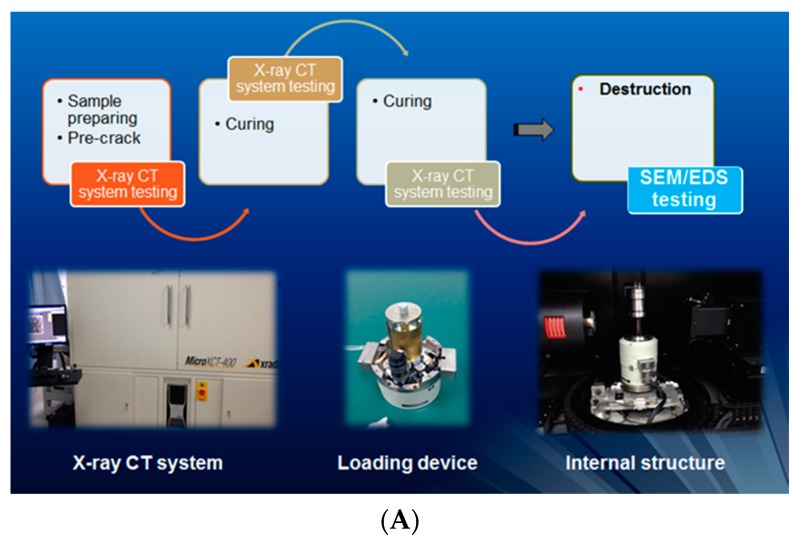

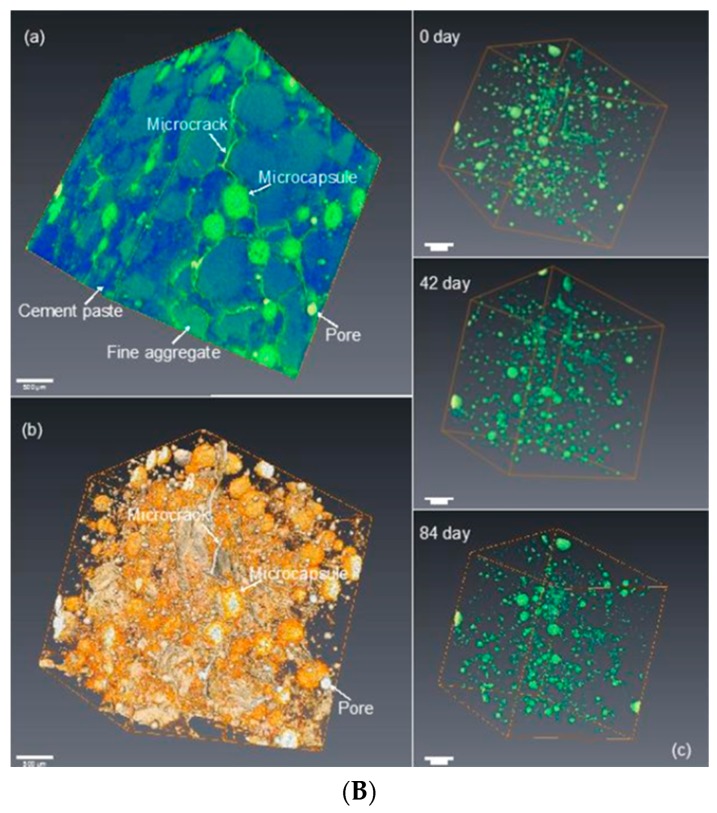
(**A**) The experimental procedure and the relevant equipment; (**B**) 3D reconstruction of self-healing sample at different healing times [49].

**Figure 22:**
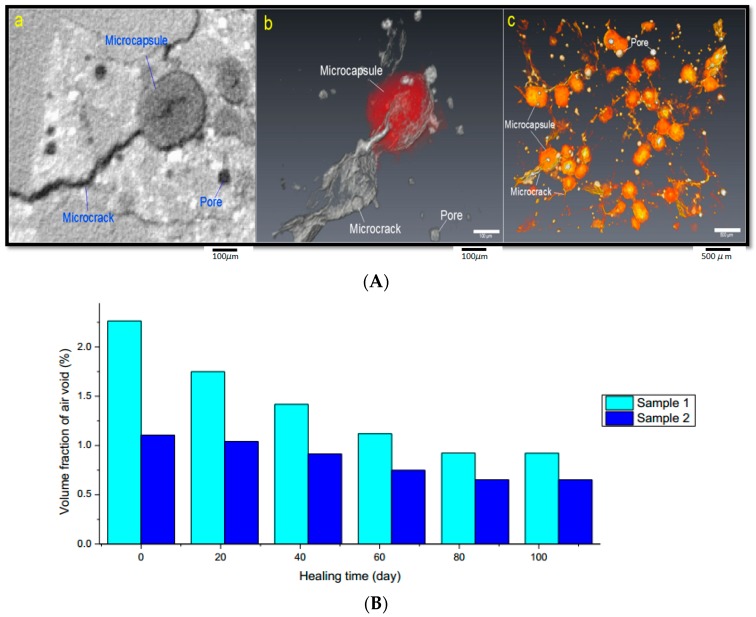
(**A**) Microcapsules triggered by cracking and subsequent healing monitored by XCT in 3D; (**B**) Quantitative evaluation of the healing effect based on the image analysis technique [49].

The whole healing process and healing effect of a microcapsule based self-healing system in concrete was monitored and qualitatively as well as quantitatively evaluated by means of XCT and an image analysis technique. The experimental procedures are illustrated in Figure 21A. A microscopic compression testing instrument was integrated into the XCT system to obtain a real-time compressive loading. Sample 1 and Sample 2 were put in the built-in loading device and pre-loaded with 1100 N and 900 N, respectively. As shown in Figure 22A, microcapsules are broken by the formation of cracks (working mechanism of a physical trigger) and healing materials are released from microcapsules. Then, the samples with initial cracks were cured in different curing conditions, in which Sample 1 was submerged and Sample 2 was cured in a standard curing condition (95% RH, ±20 °C). XCT was applied to monitor the variation of inner microstructures at different healing times (see Figure 21B). Based on the raw data measured by XCT, the reconstruction of the inner microstructure was carried out by means of an image analysis technique. 

The crack-healing process and healing effect were evaluated with the help of the reconstruction of inner microstructures (including 2D slices, 3D digital image) and quantitative analysis. With the help of an image analysis technique, the total volume of air void (cracks and pores) was labeled and calculated. As shown in Figure 22B, the total volume of air void (cracks and pores) of both samples was decreased as the healing time increased. With further calibration, the adopted method could help to evaluate the healing effect in a quantitative way.

## 5. Challenges and Further Development

The evaluation of performance characterization of self-resilience system remains to be a major challenge. Due to the inhomogeneity and randomness, bulk behavior of the concrete after resilience is difficult to experimentally evaluate at a macro level. The only feasible way to do this is to examining the individual behavior of a single unit in the randomly distributed system on a fundamental level. The relevant material properties are then determined experimentally with the help of specific facilitated test set-ups. Afterwards, numerical methods have to be exploited by means of the obtained key parameters at the micro level to simulate the bulk behavior of the resilient system.

**Figure 23:**
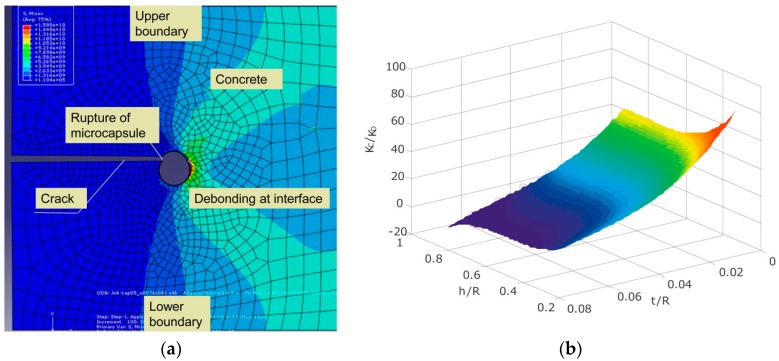
(**a**) Computing model for a crack approaching a microcapsule; (**b**) Criterion surface for determination of a microcapsule rupture or de-bonding [50].

The behavior of a single microcapsule in concrete was numerically studied at Shenzhen University [50]. The effect of the physical trigger (cracking) on mechanical behaviors of the single microcapsule was simulated, whereas the size and the thickness of microcapsule wall, the strength of the microcapsule wall, the bonding strength between the microcapsule and concrete and the direction of the crack approaching the microcapsule were considered as the key parameters (Figure 23a). The criterion surface for determination of a microcapsule rupture or de-bonding is shown in Figure 23b. A point under the surface means that a microcapsule ruptures. Otherwise, the microcapsule is de-bonded. With this criterion, the behavior of a microcapsule can be judged by using the parameters without need for FEM computation every time.

It is evident that the key parameters have to be experimentally determined numerically to obtain a realistic picture. To realize this, the following working activities have to be fulfilled: (a) working out feasible and workable micro testing facilities; (b) quantifying the relevant key parameters for various components (such as the material properties of microcapsule, healing agent, interface between microcapsule and cement matrix, static and kinetic performance of the healing process, etc.; (c) quantifying the physical and chemical trigger mechanism; and (d) examining the healing and recovery performance of a single microcapsule system.

**Figure 24:**
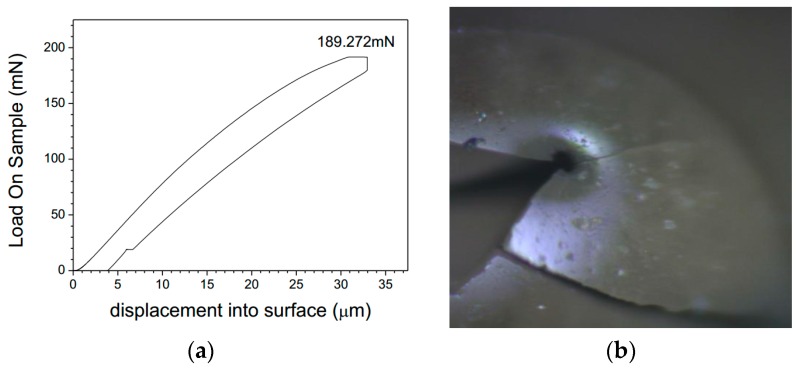
(**a**) Load-displacement curve of the microcapsule; (**b**) the image of ruptured microcapsule after test [52].

An attempt in this direction was made recently at Shenzhen University [51,52,53]. A single microcapsule with different diameter and shell thickness were selected under an optical stereoscopic microscope and then bonded on the test platform. The micromechanical properties of single microcapsule such as Load-Displacement curve, E-modulus and rupture force were obtained by a nanoindentor (see Figure 24). The elastic modulus can be indirectly calculated. However, the strength of the shell material itself needs to be further determined. The total mechanical behavior of a single microcapsule should be simulated if the correct parameters are adopted.

The evaluation of a self-resilience system plays a paramount role as far as the successful application of such a system in concrete structures is concerned. Current approaches and methodologies are strongly based on the traditional macro level methods, which make it difficult to appraise newly created systems in an appropriate way. To deal with this challenge, a new RILIEM TC SHE was set up recently (in June 2016), which aims at solve this problem through international cooperation. 

## 6. Concluding Remarks 

A comprehensive self-resilience system was developed protecting against attacks in aggressive environments, resulting in concrete structures with durable and sustainable properties that far exceed those of conventional ones. Multi-scale self-healing and self-recovery systems, embedded in concrete, will provide the required resilience. Research at Shenzhen University will focus on the following key issues to address the major challenges related to the development and application of a microcapsule based on self-resilience systems in concrete structures, namely to:

(i) establish the relationship between the multi-actions and the action effect for concrete;
(ii) link resilience requirements with environmental action and the related degradation mechanisms;
(iii) incorporate existing self-healing and self-recovery systems to enhance the long-term performance of concrete structures by creating resilience to physical and chemical attack, through the addition of agents within microcapsules, that are able to mitigate the action effects in a range of aggressive environments;
(iv) set up facilities and procedure to evaluate self-healing and self-recovery effects at the micro level;
(v) quantify key parameters related to fundamental material properties in order to supply the basic input for numerical simulations;
(vi) validate the design procedure and numerical model using data from bespoke tests at multi-scales;
(vii) demonstrate the self-resilience system by using experimental and numerical data.
(viii) realize applications in practice.
